# Evaluation of serum neurofilament light chain and glial fibrillary acidic protein in the diagnosis of Alzheimer’s disease

**DOI:** 10.3389/fneur.2024.1320653

**Published:** 2024-01-30

**Authors:** Tangni Fang, Yaqian Dai, Xueyi Hu, Yuanhong Xu, Jinping Qiao

**Affiliations:** Department of Clinical Laboratory, The First Affiliated Hospital of Anhui Medical University, Hefei, China

**Keywords:** NfL, GFAP, Alzheimer’s disease, biomarker, diagnosis

## Abstract

**Purpose:**

This study aimed to evaluate the use of serum neurofilament light chain (NfL) and glial fibrillary acidic protein (GFAP) in the diagnosis of Alzheimer’s disease (AD) and the differential diagnosis between AD and mild cognitive impairment (MCI).

**Methods:**

From September 2021 to October 2022, we collected venous blood from patients and healthy individuals who visited our hospital’s Neurology Department, and we isolated serum to detect NfL and GFAP using direct chemiluminescence. The results were analyzed using one-way analysis of variance (ANOVA) analysis and receiver operating characteristic (ROC) curves.

**Results:**

Pairwise comparisons among the three groups showed that compared with the health checkup (HC) group, serum NfL and GFAP were increased in both AD and MCI (*P*_NfL_ < 0.05, *P*_GFAP_ < 0.01). There were significant differences in GFAP between MCI and AD groups, and the level in AD group was higher (*p* < 0.01), while there was no difference in NfL. Both serum NfL and serum GFAP levels can independently diagnose AD (*p* < 0.01). The ROC curve showed that GFAP had a higher diagnostic efficacy, with an area under the ROC curve (AUC) of 0.928. The cut-off values of the two serum markers for the diagnosis of AD were NfL > 40.09 pg./mL and GFAP >31.40 pg./mL. Sensitivity and specificity for NfL in the diagnosis of AD were 59.6 and 76.2%, respectively, and for GFAP, they were 90.4 and 82.1%, respectively. The combined diagnosis of GFAP and NfL improved the diagnostic efficiency (AUC = 0.931, sensitivity = 78.8%, specificity = 92.3%). The cut-off value of GFAP for the differential diagnosis of MCI and AD was 46.05 pg./mL.

**Conclusion:**

Both serum NfL and serum GFAP can be used as biomarkers for the diagnosis of AD. Serum GFAP has better diagnostic efficacy and can distinguish AD from MCI. A combined diagnosis can improve diagnostic specificity.

## 1 Introduction

Alzheimer’s disease (AD) is the primary cause of dementia, predominantly affecting individuals over 60 years old (1). According to the World Alzheimer Report 2021, more than 55 million people worldwide suffer from dementia. As a chronic progressive neurodegenerative disease, AD is mainly characterized by progressive memory deficits, personality change, cognitive impairment and other symptoms (2, 3). The most significant risk factor for AD is advanced age (≥65 years) and carrying at least one ApoE ε4 allele (4). Pathologically, abnormal accumulation of extracellular β-amyloid protein (Aβ) and formation of neuronal fibrillary tangles by intracellular hyperphosphorylated tau protein (p-tau) are observed in patients with AD (5), which can appear up to 10–15 years before clinical onset (6). Early intervention can improve symptoms and delay disease progression. However, early diagnosis is crucial for effective intervention. Therefore, establishing convenient, economical and effective technical methods for early diagnosis of AD holds great significance in improving patients’ quality of life (7).

Available diagnostic methods include magnetic resonance imaging (MRI), positron emission computed tomography, and cerebrospinal fluid (CSF) biomarkers; however, these examinations have limitations such as being expensive or invasive (8). Blood markers are more cost-effective and time-efficient than CSF markers (9) while also providing advantages such as simple operation procedures and reduced patient discomfort, making them suitable for clinical examination or colony screening. At present, known blood markers include Aβ, p-tau, neurofilament light chain (NfL), glial fibrillary acidic protein (GFAP), etc. (10).

NfL, an intermediate filament protein exclusively expressed in neurons, constitutes one of the three subunits of neurofilaments (NFs). In a normal physiological state, NfL is released from axons at low levels in an age-dependent manner; however, during instances of inflammation, neurodegenerative diseases, trauma, or vascular injury, there is a substantial increase in NfL release (11). Moreover, plasma NfL levels exhibit a significant correlation with CSF NfL levels (12), making it a promising blood-based biomarker for neurodegeneration across various neurological disorders including AD (13, 14).

GFAP, an intermediate fiber, is expressed by astrocytes in the central nervous system and non-myelinating Schwann cells in the peripheral nervous system (15). GFAP serves as a specific biomarker for brain Aβ deposition, independent of tau protein aggregation. Astrocytosis, characterized by increased GFAP expression, is commonly observed around Aβ plaques in AD patients’ brains (16). Elevated levels of GFAP have also been detected in the serum of patients with various types of dementia, traumatic brain injury, epileptic seizure, Parkinson’s disease, multiple sclerosis, and spinal cord injury (17). However, GFAP levels are higher in AD patients compared to other conditions and can be used alongside plasma p-tau181 to differentiate between different types of dementia (18).

This study included participants from both healthy and cognitively impaired populations in Anhui province, China. The concentrations of NfL and GFAP in peripheral blood samples from patients with varying degrees of nervous system diseases were analyzed to investigate changes in their levels across different stages of disease progression, to evaluate their potential as biomarkers for AD, and to assess the diagnostic value and combined diagnostic value of blood NfL and GFAP for AD.

## 2 Materials and methods

### 2.1 Reagents and instruments

Serum NfL assay kit, serum GFAP assay kit (direct chemiluminescence method, Maccura Biotechnology Co., LTD., China), automated chemiluminescence immunoassay analyzer (Maccura Biotechnology Co., LTD., China).

### 2.2 Methods

#### 2.2.1 Participants

All participants in this study were health checkup (HC) population or patients recruited from the Department of Neurology at the First Affiliated Hospital of Anhui Medical University, China from September 2021 to October 2022. The age range was 51 to 85 years, with an average age of 66.1 ± 7.2 years. A total of 185 subjects were divided into three groups: 85 subjects in the HC group, including 41 males and 44 females, with an average age of 65.6 ± 5.3 years; 48 subjects in the mild cognitive impairment (MCI) group, including 20 males and 28 females, with an average age of 65.5 ± 9.0 years; 52 subjects in the AD group, including 24 males and 28 females, with an average age of 67.5 ± 8.0 years.

MRI was employed to assess the brain during the imaging examination. To evaluate cognitive function, the Mini-Mental State Examination (MMSE) was administered, and the severity of dementia was assessed using the Clinical Dementia Rating (CDR). Diagnosis of all patients was performed by neurologists at our hospital, based on the Chinese Classification and Diagnostic Criteria of Mental Disorders, Third Edition (CCMD-3) (19). All patients satisfied the diagnostic criteria for AD of the National Institute of Aging and Alzheimer’s Association (NIA-AA) (20). MCI is characterized by cognitive decline and degenerative brain lesions on MRI, but patients with MCI are still able to perform daily activities without dementia (CDR < 1). AD, on the other hand, is characterized by cognitive impairment, brain degeneration, and significant impairment of daily activities (CDR ≥ 1). Patients who were clinically diagnosed as cognitively normal and showed no brain degeneration on MRI were categorized as HC. Patients with comorbidities such as malignant tumors, diabetes or other psychiatric disorders have been excluded from the study. In accordance with the CCMD-3 guidelines, we will also exclude participants who possess any of the following characteristics, to eliminate the possibility of other types of dementia: cerebrovascular injury, evidence of traumatic brain injury, use of medications that cause neurological damage, secondary brain damage caused by viral infection (such as human immunodeficiency virus), Parkinson’s disease (with symptoms such as static tremor), symptoms or family history of Huntington’s disease.

#### 2.2.2 Collection of serum samples

Blood samples were collected from the elbow veins of all participants using vacuum tubes containing procoagulant and separation glue. All subjects took fasting blood samples in the morning before treatment. Samples were centrifuged at 4°C for 5 min within 30–60 min after collection to separate the serum fraction. We collected serum samples from 185 individuals and analyzed them using the following methods.

#### 2.2.3 The concentrations of NfL and GFAP were measured by direct chemiluminescence

The direct chemiluminescence method was used, based on the principle of magnetic particle chemiluminescence immunoassay and double antibody sandwich method. The magnetic particle coated with antibody, sample and acridinium ester labeled antibody were mixed and incubated to form an “antibody–antigen–antibody” complex. The unbound acridinium ester conjugate and other substances were removed by washing. The chemiluminescence reaction was performed after adding the substrate solution, and the relative light unit (RLU) was measured. RLU was proportional to the concentration of the tested substance in the sample.

The blank limit and detection limit of NfL and GFAP detection reagents were not higher than 5.0 pg./mL and 10.0 pg./mL, respectively. The intra-assay and inter-assay coefficients of variation were not higher than 6 and 10%, respectively.

### 2.3 Statistical analyses

IBM SPSS Statistics 26.0 software and GraphPad Prism 9.0 software were used for summary, statistics and data analysis. Chi-square test was employed to analyze count data. In light of the sample size exceeding 50, we employed the Kolmogorov–Smirnov (K-S) test to evaluate normality. Measurement data were represented as “x ® ± sx “and log-transformed if not normal. One-way analysis of variance (ANOVA) was conducted for group comparisons, followed by pairwise comparison using the least significant difference (LSD) test. Inter-group comparisons with uneven variances were performed using the corrected F test (Welch’s test), a significance level of *p* < 0.05 was considered statistically significant. Binary logistic regression was utilized to examine the correlation between disease and serum concentrations of NfL and GFAP, when *p* < 0.05 is achieved, the covariate is considered as the risk factor of the dependent variable. The Hosmer-Lemeshow (HL) test was conducted to assess the goodness of fit. Specifically, a model fitted well when the *p*-value was greater than 0.05. Receiver operating characteristic (ROC) curve analysis was employed to evaluate and compare the diagnostic value of each biomarker. It is generally believed that the area under the ROC curve (AUC): greater than 0.9 indicates high diagnostic accuracy, between 0.7 and 0.9 indicates moderate diagnostic accuracy, and between 0.5 and 0.7 indicates low diagnostic accuracy. Significance level *α* = 0.05, *p* < 0.05 indicates that the difference is statistically significant. At the same time, the optimal diagnostic cut-off values for each serum biomarker were obtained. In order to reduce false positives, we used the Benjamini-Hochberg (BH) test to correct the *p*-value of the entire paper. The evaluation indexes were sensitivity and specificity. The predictive values were positive predictive value (PPV) and negative predictive value (NPV). The comprehensive evaluation indexes were Youden index, positive likelihood ratio (LR+) and negative likelihood ratio (LR-).

## 3 Results

### 3.1 The classification of etiology

From September 2021 to October 2022, patients who underwent physical examination and medical treatment at the First Affiliated Hospital of Anhui Medical University were collected and divided into three cohorts, including 85 cases in HC group, 48 cases in MCI group and 52 cases in AD group. Table 1 displays the distribution of age and gender, along with the MMSE test results. The measured data followed a normal distribution. The gender comparison was done using the χ2 test, while the age comparison was done using the corrected F-test due to unequal variance. No significant differences were found in age and gender (*p* < 0.05). An independent sample t-test was used to compare the MMSE scores, which showed significant differences between the MCI and AD groups (*p* < 0.01).

**Table 1:** Basic information of the participants.

Groups	n	Gender (M/F)	Age	MMSE
HC	85	41/44	65.6 ± 5.3	–
MCI	48	20/28	65.5 ± 9.0	21.7 ± 4.9
AD	52	24/28	67.5 ± 8.0	12.8 ± 6.3
*F*/*χ^2^*/*Z*	–	0.534	1.266	2.016
*P*	–	0.766	0.284	< 0.01

– means no date. HC, health check. MCI, mild cognitive impairment. AD, Alzheimer’s disease. M/F, male/female. MMSE, Mini-Mental State Examination.

### 3.2 Comparison of concentrations of serum NfL and GFAP in different groups

**Figure 1:**
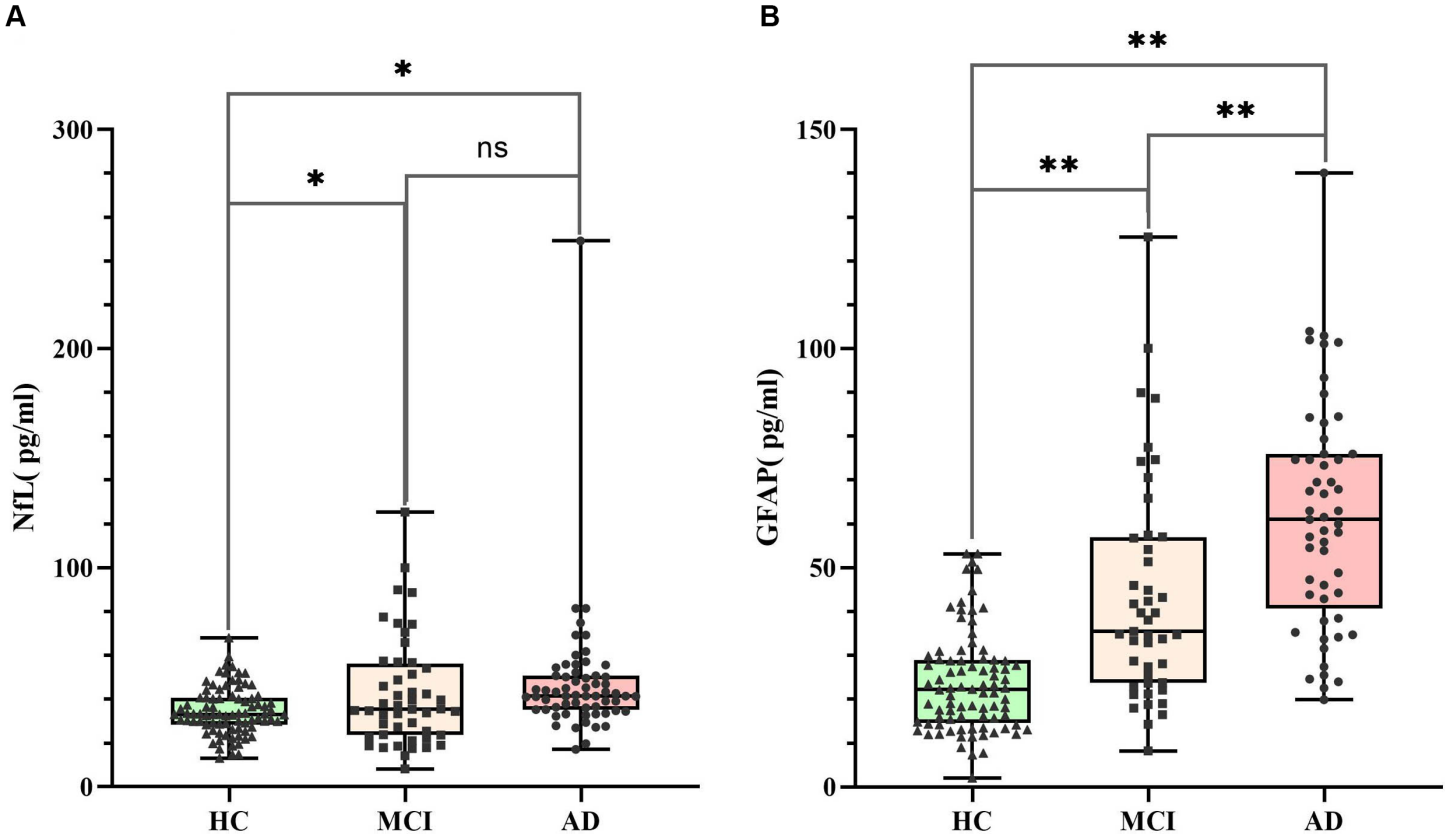
Serum NfL and GFAP levels in HC (*n* = 85), MCI (*n* = 48) and AD (*n* = 52). Panel **(A)** represents the serum NfL level of the three groups. The concentrations were 34.69 ± 1.18  pg./mL (HC), 44.87 ± 5.76  pg./mL (MCI) and 47.54 ± 4.36  pg./mL (AD), respectively. Panel **(B)** represents the serum GFAP level of the three groups. The concentrations were 23.76 ± 1.23  pg./mL (HC), 41.31 ± 3.31  pg./mL (MCI) and 63.53 ± 3.67  pg./mL (AD), respectively. Individual dots represent data obtained from individual subjects. The measurement data were analyzed by ANOVA after logarithmic transformation. Ns means the difference was not statistically significant. *p < 0.05, **p < 0.01.

The K-S test indicated a non-normal distribution of serum NfL and GFAP levels, requiring logarithmic transformation prior to ANOVA analysis. ANOVA test revealed differences in NfL and GFAP among the three groups (*p* < 0.05), and the serum NfL and GFAP levels of the three groups were compared, respectively, by posterior comparisons (LSD test; shown in Table 2). The serum NfL concentration and serum GFAP concentration of the three subject groups were plotted in a box diagram (shown in Figure 1), the group was taken as the abscissa coordinate, and the serum marker concentration was taken as the ordinate coordinate. Figure 1A compares serum NfL concentration among the three groups of subjects, and Figure 1B compares serum GFAP concentration among the three groups of subjects. Serum NfL levels in both MCI and AD groups were higher than those in HC group, and the difference was statistically significant (*p* < 0.05), but there was no difference between the MCI and AD groups (*p* > 0.05). Serum GFAP levels in the MCI and AD groups were significantly different from those in the HC group, and GFAP could distinguish between MCI and AD (*p* < 0.01). These results indicate that serum NfL and GFAP can be used as diagnostic biomarkers for MCI and AD, and GFAP can be used as differential diagnostic biomarkers for MCI and AD.

**Table 2:** Serum levels of the two indicators were compared among the participants in each group.

Groups	n	NfL (pg/ml)	GFAP (pg/ml)
HC	85	34.69 ± 1.18	23.76 ± 1.23
MCI	48	44.87 ± 5.76^a^	41.31 ± 3.31^b^
AD	52	47.54 ± 4.36^a,c^	63.53 ± 3.67^b,d^

*P*^a^ < 0.05, *P*^b^ < 0.01, vs. HC group; *P*^c^ > 0.05, *P^d^* < 0.01, vs. MCI group.

### 3.3 ROC curve analysis

Logistic regression analysis indicated that both serum NfL and GFAP were risk factors for MCI/AD diagnosis (*p* < 0.05; shown in Table 3). The concentration of GFAP in serum was found to be more strongly associated with the disease than the concentration of NfL, according to their respective OR values. The HL test yielded a *p* > 0.05, indicating a satisfactory fit of the logistic regression model. Linear regression analysis was performed on the independent variables, and collinearity diagnosis indicated that the variables were not subject to multicollinearity (VIF < 2). Therefore, ROC analysis was conducted for NfL and GFAP (shown in Table 4).

**Table 3:** Screening of risk factors for MCI and AD.

Groups	MCI		AD	
	OR (95% *CI*)	*P*	OR (95% *CI*)	*P*
NfL	1.024 (0.999, 1.049)	0.041	1.065 (1.030, 1.101)	0.000
GFAP	1.070 (1.039, 1.103)	0.000	1.126 (1.082, 1.170)	0.000

OR, odds ratio. CI, confidential interval.

**Table 4:** The area under the one-factor characteristic ROC curve of HC and MCI, HC and AD.

Groups		AUC	Standard error	*P* (adjusted *P*)	Approaching 95% *CI*	
					Minimal value	Maximum value
MCI	NfL	0.577	0.055	0.150 (0.150)	0.470	0.684
	GFAP	0.706	0.044	0.000 (0.000)	0.673	0.847
	NfL + GFAP	0.776	0.044	0.000 (0.000)	0.690	0.861
AD	NfL	0.708	0.046	0.002 (0.002)	0.619	0.798
	GFAP	0.928	0.022	0.000 (0.000)	0.886	0.971
	NfL + GFAP	0.931	0.021	0.000 (0.000)	0.890	0.973

CI, confidential interval. AUC, the area under the ROC curve.

**Figure 2:**
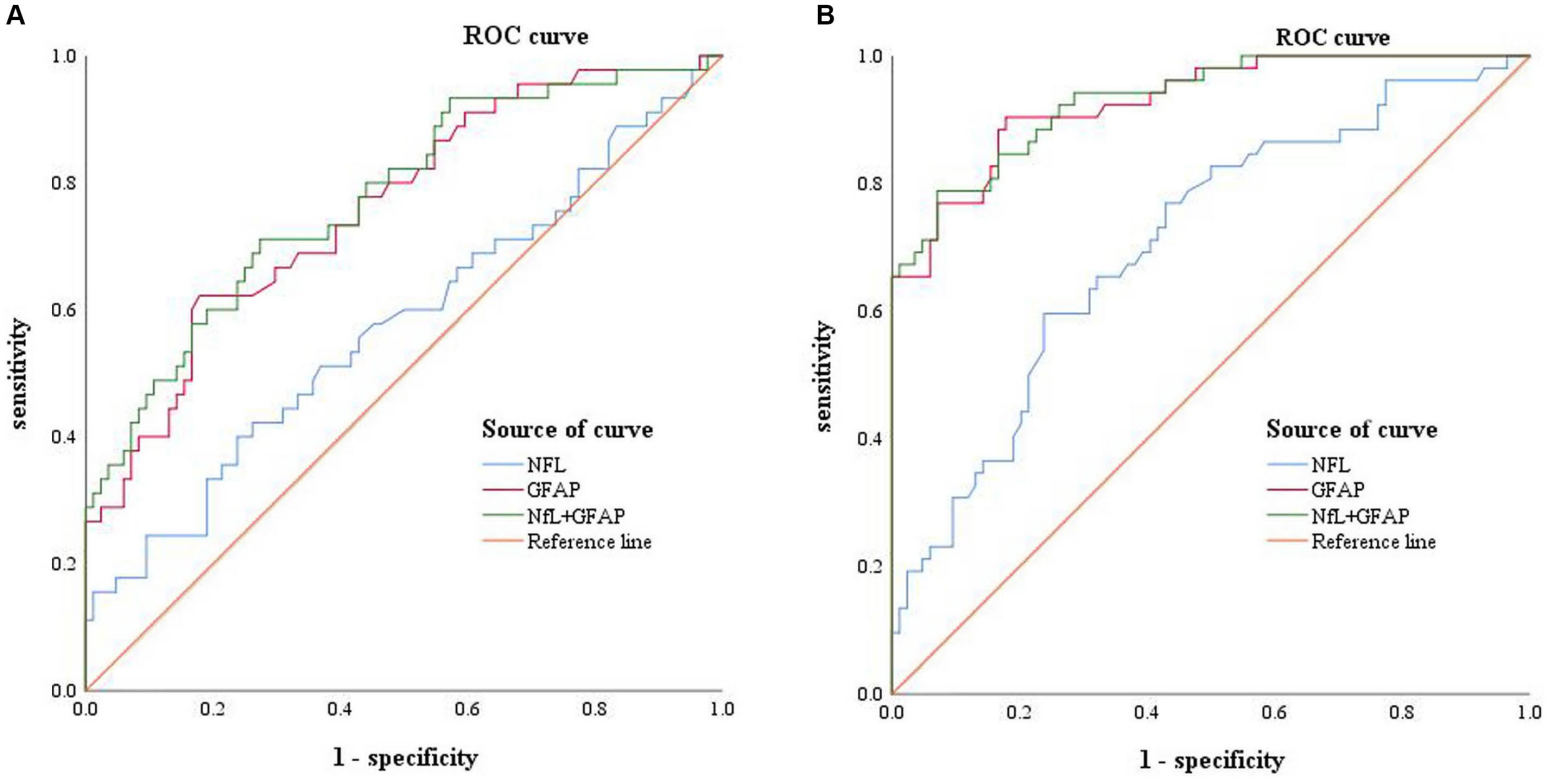
Receiver operating characteristic curve: Panel **(A)** represents the ROC curve of MCI patients, including NFL (blue, AUC  =  0.577), GFAP (red, AUC  =  0.706), combined diagnosis (green, AUC  =  0.776), and reference line (orange). Panel **(B)** represents the ROC curves of AD patients, including NFL (blue, AUC  =  0.708), GFAP (red, AUC  =  0.928), combined diagnosis (green, AUC  =  0.931), and reference line (orange).

The ROC curves of MCI and AD were plotted with sensitivity as the ordinate coordinate and 1-specificity as the abscissa coordinate (shown in Figure 2), AUC and Yoden index were calculated, and the diagnostic efficacy of serum NfL and GFAP for MCI and AD were analyzed. The results indicated that NfL alone could not diagnose MCI. Serum GFAP was found to be the more effective single factor in diagnosing AD, with an AUC of 0.928 and a maximum Yoden index of 0.725. The optimal cut-off value for diagnosing AD (i.e., NfL > 40.9 pg./mL, GFAP >31.40 pg./mL), sensitivity, specificity, predicted value, likelihood ratio and other indicators of single detection and combined detection of each indicator can be obtained (shown in Table 5); among these indicators, serum GFAP has higher sensitivity and specificity while combined diagnosis improves the specificity of AD diagnosis.

**Table 5:** Clinical evaluation of MCI and AD diagnosis results of NFL and GFAP.

Groups		Youden index	Cut-off value	Se (%)	Sp (%)	PPV	NPV	LR+	LR–
MCI	NfL	0.162	40.85	40.0	76.2	0.49	0.69	1.68	0.79
	GFAP	0.444	32.05	62.2	82.1	0.66	0.79	3.48	0.46
	NfL + GFAP	0.437	–	71.1	72.6	0.59	0.82	2.60	0.40
AD	NfL	0.358	40.9	59.6	76.2	0.61	0.76	2.50	0.53
	GFAP	0.725	31.40	90.4	82.1	0.76	0.93	5.06	0.12
	NfL + GFAP	0.717	–	78.8	92.3	0.86	0.88	11.04	0.23

– means no date. Se, sensitivity. Sp, specificity. PPV, positive predictive value. NPV, negative predictive value. LR, likelihood ratio.

A Delong test was conducted to compare the differences between the three AUC values (shown in Table 6). The results indicated a significant difference in the AUC between NfL and GFAP when diagnosing MCI and AD, as well as between NfL and the combined diagnosis (*p* < 0.01). However, there was no significant difference in AUC between GFAP and combined diagnosis in either MCI or AD diagnosis (*p* > 0.05).

**Table 6:** Comparison of AUC values of serum markers for the diagnosis of MCI and AD.

Groups		*Z*	*P* (adjusted *P*)	AUC difference	Approaching 95% *CI*	
					Minimal value	Maximum value
MCI	GFAP vs. NfL	2.926	0.003 (0.004)	0.183	0.061	0.306
	NfL + GFAP vs. NfL	3.844	0.000 (0.000)	0.199	0.097	0.300
	NfL + GFAP vs. GFAP	0.748	0.454 (0.545)	0.015	0.025	0.056
AD	GFAP vs. NfL	4.789	0.000 (0.000)	0.220	0.130	0.310
	NfL + GFAP vs. NfL	5.276	0.000 (0.000)	0.223	0.140	0.306
	NfL + GFAP vs. GFAP	0.498	0.618 (0.618)	0.003	0.009	0.015

CI, confidential interval. AUC, the area under the ROC curve.

### 3.4 Differential diagnosis of serum biomarkers for MCI and AD

**Figure 3:**
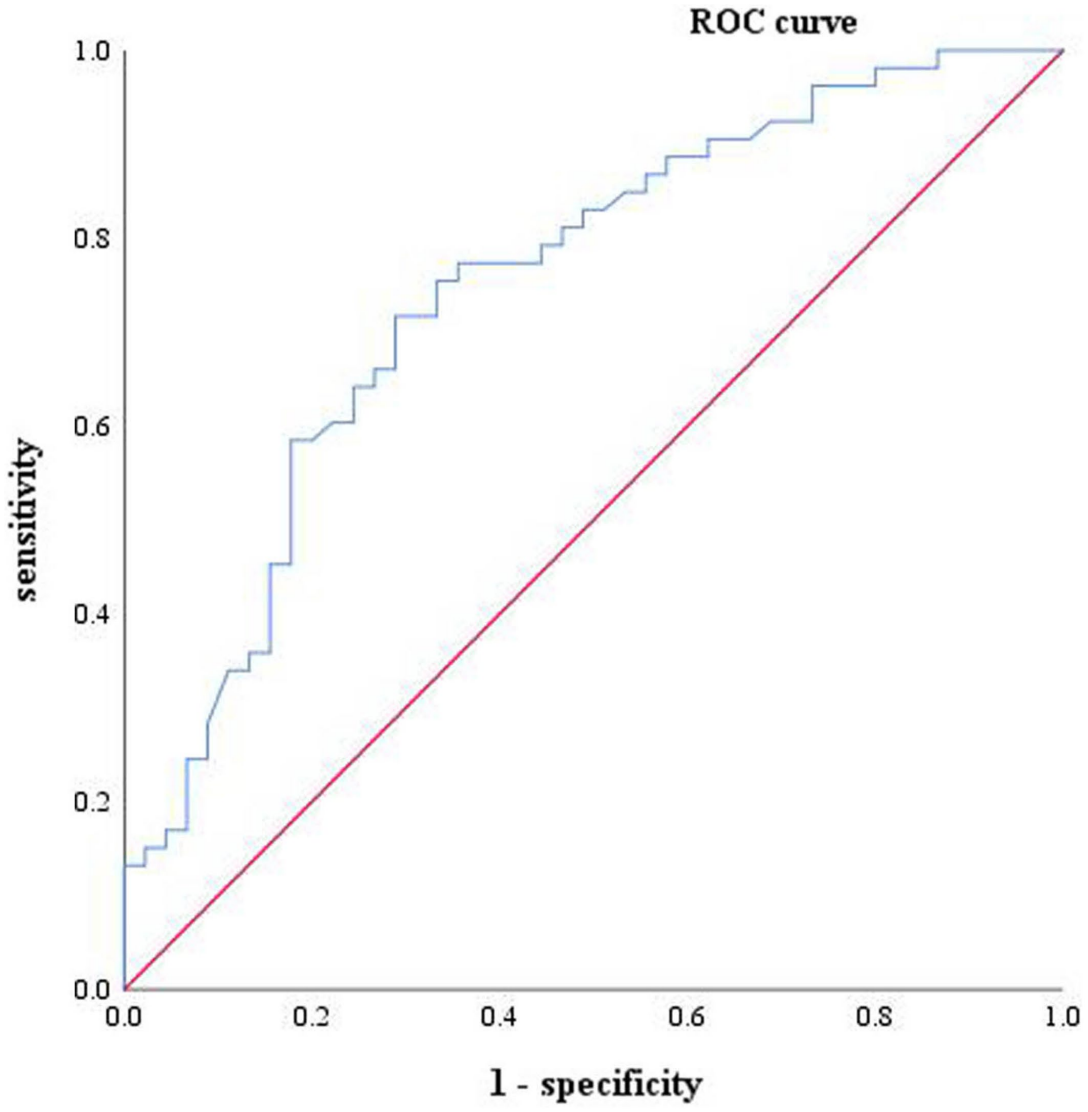
One-factor characteristic ROC curve of AD and MCI, including GFAP (blue, ACU = 0.749) and reference line (red).

Based on these findings, it can be concluded that only serum GFAP effectively differentiates AD from MCI. Therefore, the following analysis is made to identify MCI and AD by serum biomarkers. Logistic regression analysis was performed for the two markers (shown in Table 7). ROC analysis was performed on GFAP and the ROC curve was drawn (shown in Figure 3). The results of the calculation showed that the AUC was 0.749 (0.651 ~ 0.846), the sensitivity was 71.7%, the specificity was 71.1%, and the Yoden index was 0.428. The PPV, NPV, LR+ and LR- were 0.73, 0.70, 2.48, and 0.40, respectively. The cut-off value of 46.05 pg./mL was found to be the best critical value for the differential diagnosis of MCI and AD.

**Table 7:** Identification factor screening of MCI and AD.

Biomarker	OR (95% *CI*)	*P*
NfL	1.002 (0.991, 1.014)	0.708
GFAP	1.039 (1.018, 1.059)	0.000

OR, odds ratio. CI, confidential interval.

## 4 Discussion

Astrocytes, the most widely distributed type of cells in the mammalian brain, are activated in response to neurological diseases (21). In AD, astrocytes play a crucial role in inflammatory processes, amyloid clearance, and neurovascular coupling (22). GFAP, a significant component of the astrocyte cytoskeleton, serves as a marker for astrocyte activation and can identify astrocytosis in the early stage of AD (16, 23). In AD cases, GFAP can reflect the response of astrocytes to Aβ deposition in the brain (24), with plasma GFAP showing a stronger correlation with brain Aβ pathology than CSF GFAP (16). NfL, an intermediate filament protein released by neuronal axons, is one of three subunits of NFs. It serves as a biomarker for nerve damage. Although the specific function of NFs remains unclear, they contribute to radial growth promotion and maintenance of axon stability (13). As a marker for axonal injury, NfL alone cannot distinguish neurological diseases with similar pathological changes.

The levels of serum GFAP in HC, MCI and AD groups were 23.76 ± 1.23 pg./mL (HC), 41.31 ± 3.31 pg./mL (MCI) and 63.53 ± 3.67 pg./mL (AD), respectively. The NfL concentration was 34.69 ± 1.18 pg./mL (HC), 44.87 ± 5.76 pg./mL (MCI) and 47.54 ± 4.36 pg./mL (AD), respectively. The ANOVA results of this study indicate that compared to HC group, the elevated levels of blood NfL and GFAP in patients with MCI and AD are statistically significant (*P*_NfL_ < 0.05, *P*_GFA*p*_ < 0.01); thus suggesting their potential use as diagnostic markers. However, only GFAP can differentiate between MCI and AD cases (*P* < 0.01), while there was no statistically significant difference in NfL concentration between the two groups (*p* > 0.05). In AD patients specifically, reactive astrocytes proliferate leading to increased release of GFAP into the bloodstream. Numerous studies have demonstrated higher levels of blood GFAP in AD patients compared to other neurodegenerative diseases due to variations in neuroinflammation heterogeneity or different types/patterns of astrocyte hyperplasia observed across various neurodegenerative conditions (17). In the case of NfL, when axons are injured or damaged, NFs are released from neuronal axons into extracellular spaces within cells before entering CSF, and into the bloodstream after crossing the blood–brain barrier. This allows for the detection of NfL in peripheral blood (13). Multiple lines of evidence have demonstrated elevated levels of blood NfL in AD patients with certain predictive values (25, 26). Therefore, increased levels of both blood GFAP and NfL can be detected during neurodegenerative diseases such as AD, which is consistent with the findings of this study.

ROC analysis revealed that both NfL and GFAP were risk factors for MCI and AD (*p* < 0.05). The cut-off values for the diagnosis of AD were NfL > 40.09 pg./mL and GFAP >31.40 pg./mL, respectively. While GFAP can serve as a diagnostic biomarker for MCI and AD, NfL alone cannot diagnose MCI (AUC = 0.577, *p* > 0.05). Moreover, the NfL biomarker was found to be insufficient in distinguishing between MCI and AD, which is consistent with the results of the ANOVA. This finding is in contrast to prior research. A meta-analysis of studies has indicated that AD patients exhibit higher levels of NfL in serum in comparison to MCI patients (27). The combination of serum NfL and GFAP was used in the diagnosis of AD. ROC analysis showed that the specificity (92.3%) and diagnostic efficacy (AUC = 0.931) of combined diagnosis were higher than those of single markers. GFAP may also hold potential as a biomarker for other types of dementia since astrocytes are not specific to AD pathophysiology (28). For instance, astrocyte dysfunction occurs in neurodegenerative diseases such as Parkinson’s disease, amyotrophic lateralizing sclerosis, and Huntington’s disease (29). However, higher levels of GFAP were observed in dementia cases. Previous studies have reported that cognitively healthy older adults at risk for cognitive impairment had higher blood concentrations of GFAP compared to control participants; moreover, elevated blood concentrations were associated with cognitive decline and dementia (17), supporting our study’s conclusions. A study has revealed that GFAP is an autonomous predictor of memory performance and has a negative correlation with memory (30). Several studies have shown that plasma NfL levels are elevated in both frontotemporal dementia (FTD) and AD; however, it lacks specificity and has limited ability to distinguish between FTD subtypes or between FTD and AD (25, 31, 32). Plasma NfL outperforms plasma total tau protein (t-tau) in diagnosing FTD and AD while exhibiting a strong correlation with CSF NfL (33). The study conducted by Mattsson N et al. demonstrated that blood levels of NfL were higher in individuals with MCI compared to healthy people (25). However, it should be noted that elevated levels of NfL are also observed in other conditions characterized by axonal damage to neurons. The review revealed that the non-specific elevation of NfL was found in nerve injury diseases beyond dementia, while GFAP was more relevant to Aβ deposition (10). Consistent with these findings, our results indicate that only GFAP can differentiate between MCI and AD. The best cut-off value of GFAP to distinguish MCI from AD was 46.05 pg./mL, that is, higher than this value can be diagnosed as AD. In summary, the results of ROC analysis in this paper showed that GFAP had a strong diagnostic efficacy for AD (AUC = 0.928), indicating superior specificity and diagnostic efficiency compared to serum NfL. Although the AUC value of combined diagnosis was higher than that of GFAP, the Delong test showed no meaningful difference. Hence, GFAP alone is enough in practical applications.

It is worth noting that this study’s findings differ from those of some previous studies. Specifically, NfL was unable to differentiate between AD and MCI. One possible explanation for this discrepancy could be the limited sample size of this study, which was restricted to one region and may not be representative of other populations. Additionally, it is important to consider that MCI can present differently in different patients and can be categorized into various subtypes based on clinical symptoms. These subtypes include amnestic MCI, multidomain MCI (amnestic or non-amnestic), and single non-memory MCI. The causes of MCI can also vary greatly and may include degenerative, vascular, metabolic, traumatic, psychiatric, and other factors (34). The study conducted by Shim confirmed that serum NfL varied in different types of MCI (35). However, the study did not provide a detailed breakdown of the different groups of MCI symptoms and causes. As mentioned earlier, elevated levels of NfL are not specific to MCI but can also be seen in other diseases with neuronal axonal damage. This is one of the reasons why the elevation of serum NfL in MCI patients was not significantly different from that in AD patients in the present study. Therefore, serum GFAP may be a better indicator of cognitive impairment than NfL when MCI is diagnosed by physicians, especially when the type of MCI is not distinguished. In fact, two studies have shown that plasma NfL cannot differentiate between MCI and AD without further explanation (36, 37).

A limitation of this study is that, apart from a detailed grouping of MCI, we did not measure cerebrospinal fluid markers or PET of brain Aβ plaques. Classical biomarkers such as Aβ, t-tau, and p-tau can be invasive and expensive (38, 39), while serum NfL and GFAP do not have these disadvantages. They can assist in diagnosing AD, but they are not specific to AD. For instance, NfL is also elevated in FTD, and hence cannot be used alone to diagnose AD. A combination of these two markers or other diagnostic methods is required to diagnose AD accurately. It is important to note that our study did not consider the participants’ APOE4 status and educational level due to cost constraints. These two factors have been identified as significant confounders of AD by previous studies (40, 41). The diagnostic criteria we used (CCMD-3) are from 20 years ago and have certain limitations, although this criterion was developed with reference to the Diagnostic and Statistical Manual of Mental Disorders (DSM-IV) and the International Classification of Diseases (ICD-10) (19). These are some of the limitations of this study that must be acknowledged.

In conclusion, both serum NfL and GFAP can serve as potential biomarkers for diagnosing AD. However, it is worth noting that serum NfL indicates neuron destruction and may not be as reliable a marker for AD as serum GFAP. Conversely, serum GFAP has been found to be closely associated with Aβ deposition, and its levels are significantly higher in AD patients, allowing differential diagnosis between MCI and AD. The combined detection of NfL and GFAP can improve the specificity of AD, resulting in more precise and reliable diagnostic outcomes.

## Data availability statement

The raw data supporting the conclusions of this article will be made available by the authors, without undue reservation.

## Ethics statement

The studies involving humans were approved by the Committee on Medical Ethics of the First Affiliated Hospital of Anhui Medical University. The studies were conducted in accordance with the local legislation and institutional requirements. The Committee on Medical Ethics of the First Affiliated Hospital of Anhui Medical University waived the requirement of written informed consent for participation from the participants or the participants’ legal guardians/next of kin since the medical records and biospecimens were obtained from previous clinical visits.

## Author contributions

TF: Data curation, Methodology, Writing – original draft. YD: Data curation, Methodology, Writing – original draft. XH: Data curation. YX: Conceptualization, Writing – review & editing. JQ: Conceptualization, Writing – review & editing.
